# Integrated transcriptome and proteome analysis reveals molecular responses of soybean anther under high-temperature stress

**DOI:** 10.3389/fpls.2023.1187922

**Published:** 2023-06-14

**Authors:** Jiajia Li, Linying Chen, Xianguan Zhi, Jianxin Wang, Yun Lu, Zhuo Tian, Meiyan Wu, Yajing Shan, Haoran Chen, Wei Liao, Qun Long, Shangshang Zhu, Juntao Wu, Lijuan Qiu, Xiaobo Wang

**Affiliations:** ^1^ School of Agronomy, Anhui Agricultural University, Hefei, China; ^2^ Institute of Crop Sciences, Chinese Academy of Agricultural Sciences/National Key Facility for Crop Gene Resources and Genetic Improvement/Key Laboratory of Soybean Biology in Beijing, Ministry of Agriculture and Rural Affairs, Beijing, China

**Keywords:** soybean, high-temperature stress, RNA-Seq, proteomics, molecular mechanism

## Abstract

It is well documented that high temperature (HT) severely affects the development of soybean male reproductive organs. However, the molecular mechanism of thermo-tolerance in soybean remains unclear. To explore the candidate genes and regulatory mechanism of soybean response to HT stress and flower development, here, the anthers of two previously identified HT-tolerant (JD21) and HT-sensitive (HD14) varieties were analyzed by RNA-seq. In total, 219 (172 upregulated and 47 downregulated), 660 (405 upregulated and 255 downregulated), and 4,854 (2,662 upregulated and 2,192 downregulated) differentially expressed genes (DEGs) were identified between JD21 anthers treated with HT stress vs. JD21 anthers in the natural field conditions (TJA vs. CJA), HD14 anthers treated with HT stress vs. HD14 anthers in the natural field conditions (THA vs. CHA), and JD21 vs. HD14 anthers treated with HT stress (TJA vs. THA), respectively. The results showed that there were more DEGs upregulated in JD21; this might be the reason why JD21 was more HT-resistant than the HT-sensitive variety HD14. GO annotation and KEGG enriched analysis showed that many DEGs are mainly involved in defense response, response to biological stimuli, auxin-activated signaling pathway, plant hormone signal transduction, MAPK signaling pathway-plant, starch and sucrose metabolism, etc. The conjoint analysis of RNA-seq and previous iTRAQ results found that there were 1, 24, and 54 common DEGs/DAPs showing the same expression pattern and 1, 2, and 13 common DEGs/DAPs showing the opposite pattern between TJA vs. CJA, THA vs. CHA, and TJA vs. THA at the protein and gene level, respectively, among which HSPs, transcription factor, GSTU, and other DEGs/DAPs participated in the response to HT stress and flower development. Notably, the qRT-PCR analysis and physiological index change results coincided with the sequencing results of RNA-seq and iTRAQ. In conclusion, the HT-tolerant cultivar performed better under stress than the HT-sensitive cultivar through modulation of HSP family proteins and transcription factors, and by keeping key metabolic pathways such as plant hormone signal transduction normal. This study provided important data and some key candidate genes to better study the effect and molecular basis of HT on anther in soybean at a transcription and translation level.

## Introduction

Temperature is a key environmental factor regulating plant growth and development, affecting crop yield formation and quality characteristics (Zhao et al., 2017a). In recent years, global temperatures have continued to rise as a result of population growth and greenhouse gas emissions, according to the Intergovernmental Panel on Climate Change: A temperature rise of 4°C will lead to extreme weather, which will affect the growth and development of crops (Chen et al., 2019). The high temperature (HT) and heat damage in the summer of 2003 reduced the national rice yield to the lowest point in nearly 20 years (Li et al., 2004; Tian et al., 2009). Climate change plays an important role in dry matter distribution, transpiration, photosynthesis, respiration, and root growth during crop growth and development. Without considering additional factors, such as precipitation, fertilization, and field management, an increase in the global average temperature of 1°C is predicted to result in 6.0%, 3.2%, 7.4%, and 3.1% reductions in yields of wheat, rice, maize, and soybean, respectively (Zhao et al., 2017a).

Soybean is an important cash crop, which can provide rich nutrients such as oil and protein for humans and animals (Li et al., 2014b), but it is sensitive to changes in sunshine length and temperature conditions (Li et al., 2020). HT stress caused by climate change has endangered all stages of soybean growth and development and has become one of the important factors restricting its yield and quality (Liu et al., 2019). In recent years, soybean crops in the Huang-Huai-Hai region of China have frequently encountered continuous HT episodes during the flowering period, which is a crucial factor underlying large-scale yield reduction (Li et al., 2020). Flowering time is of vital importance in plant development, adaptation to the environment, yield, and stress resistance. Exposure to HT stress is most harmful to crops in the flowering season (Fang et al., 2017). The flowering stage of the soybean life cycle is an important phase to attain sustainable yields as this stage is highly susceptible to HT stress (Li et al., 2020). HT seriously damages the integrity of cell membrane and reduces photosynthetic rate and respiratory rate (Xu et al., 2021), and the pollen vitality and pod setting rate will be reduced under HT stress (Muhammad et al., 2018), resulting in inevitably reducing the quality and yield of crops.

Exploration and analysis of the molecular mechanism of HT tolerance in soybean, clarification of the regulatory networks responsive to HT stress, and functional analysis of genes and proteins associated with HT stress are important to promote molecular breeding for soybean thermotolerance and cultivar development (Li et al., 2020). *SRL10* interacts with catalase isoenzyme B (CATB) to enhance the scavenging capacity of hydrogen peroxide (H_2_O_2_), thereby improving rice heat tolerance (Wang et al., 2023). To improve the HT tolerance of soybean at the molecular level, it is necessary to explore the key genes or proteins regulating HT stress. At present, transferomics and proteomics methods have been widely used to study the resistance of plants to abiotic stresses (such as HT, drought, and salt stress). Transcriptome is a powerful tool for comprehensively studying the alterations of genes caused by environmental factors and regulating metabolic pathways (Zhang et al., 2010). Raju et al. (2020) found that 1,137 genes were upregulated in sugarcane after HT stress by transferomics analysis, and ferredoxin-dependent glutamate synthase and stress protein DDR-48 were significantly induced by HT. Transcriptome analysis and genome-wide association studies (GWAS) of anthers from 218 cotton germplasm grown under HT stress identified three heat tolerance-associated motifs and provided 13,132 transcripts for expressed quantitative trait motifs (eQTL), and it also confirmed that HT stress disrupted the expression profile of *GhHRK1*, resulting in loss of pollen vitality in cotton anthers, and the results suggested that *GhHRK1* negatively regulates cotton in response to heat stress (Ma et al., 2021). Proteomics is a powerful tool for quantitative analyses of biochemical pathways, including plant stress-related responses (Lin et al., 2016). For instance, proteomics based on iTRAQ marker technology has been widely used to investigate HT stress-response mechanisms in organs of diverse crops, such as the wheat kernel (Zhang et al., 2018), rice grain (Timabud et al., 2016), tomato seedling (Sang et al., 2017), *Pyropia haitanensis* (Shi et al., 2017), and soybean anther (Li et al., 2020). In general, the transcriptome and proteome reflect gene expression at two different levels, respectively, and the integration analysis of transcriptomics and proteomics has become an inevitable trend in biological research, because they can complement each other and decipher more comprehensive molecular characteristics.

Therefore, we tried to find important genes and metabolism pathways might be related to HT resistance through the comparative transcriptome analysis between the anthers of HT-tolerant (“Jidou 21, JD21”) and HT-sensitive (“Hedou 14, HD14”) cultivars using the Illumina sequencing technology in this study. Furthermore, the conjoint analysis of RNA-Seq results and the reported iTRAQ results between JD21 and HD14 (Li et al., 2020) were performed. The aim of this study is to characterize the molecular response of soybean anthers to HT and to identify genes or proteins constitutively expressed at a higher or lower level in the cultivars for further clarifying the mechanisms involved in efficient alleviation of the adverse effects of HT stress. The results will contribute to elucidation of the molecular mechanisms underlying the response of soybean to HT stress and provide genetic resources for improvement of heat tolerance traits in soybean.

## Materials and methods

### Plant materials and growth conditions

The soybean cultivars “Jidou 21” (JD21; HT-tolerant) and “Hedou 14” (HD14; HT-sensitive) were grown in 30 cm × 40 cm × 20 cm plastic pots in 2019 at the Nongcui Garden Experimental Station of Anhui Agricultural University, China. The HT environment was created by artificial warming (by mulching with plastic greenhouse film) in the field at the flowering stage (Li et al., 2020). A temperature and humidity data logger (RC-4HC, Elitech, Berkhamstead, UK) was used to monitor the temperature stress for 7 days (09:00–16:00 on each day). Plant anther samples were collected the next morning after HT stress treatment, and the control plants anther samples were collected at the same time in the natural field environment. Anthers from control and HT-treated plants were collected in 2-ml cryopreservation tubes and stored in liquid nitrogen, and then transferred to a −80 °C medical cryopreservation box for storage. The samples used for transcriptome sequencing and iTRAQ detection were the same batch of samples. The samples from anthers of HD14 after HT stress treatment (THA), anthers of the HD14 control group (CHA), anthers of JD21 after HT stress treatment (TJA), and anthers of the JD21 control group (CJA), and transcriptome and proteome datasets from a total of 12 libraries with three biological replicates were used.

### Total RNA extraction, cDNA library construction, and Illumina-sequencing

After total RNA was extracted and DNA was digested by DNase, eukaryotic mRNA was enriched by magnetic beads with Oligo (dT) (for prokaryotes, rRNA was removed by a kit to enrich mRNA, adding the interrupt reagent to break the mRNA into short fragments). Using the interrupted mRNA as template, one-strand cDNA was synthesized with six-base random primers, and then two-strand cDNA was synthesized with the two-strand reaction system, and the double-strand cDNA was purified with the kit. The purified double-stranded cDNA was then repaired by adding an A-tail and connected to the sequencing connector, and then the fragment size was selected, and finally PCR was amplified. The constructed libraries were tested with Agilent 2100 Bioanalyzer and sequenced with Illumina HiSeq™ 2500 or Illumina HiSeq X Ten to generate 125-bp or 150-bp double-ended data. Illumina sequencer was used for sequencing after quality inspection.

### Analysis of differentially expressed genes

When using RNA-Seq data to compare and analyze whether there is differential expression of the same gene in two samples, two criteria can be selected: one is fold change (FC), which is the change multiple of the same gene expression level in two samples; the second is *p-*value or FDR (adjusted *p-*value). The calculation method of FDR value should first calculate the *p-*value of each gene, and then use FDR error control method to correct the *p-*value by multiple hypothesis test. “FDR (false discovery rate) ≤0.05 and |Log_2_FC| ≥1” were used as the threshold for judging the significance of gene expression difference.

### The conjoint analysis of RNA-Seq and iTRAQ results

For conjoint analysis of DAPs and DEGs, the transcriptomic dataset was extracted and compared with the annotated iTRAQ database (Li et al., 2020). First, the results of transcriptome sequencing were used for coding sequence prediction, and the protein sequences of these genes were obtained. Then, the predicted protein sequences were used to search the database. To avoid the occurrence of false positives, the results of transcriptome and proteome analyses were unified by searching the same database (soybean reference genome, version *Glyma 2.0*). We set “FDR ≤ 0.05, | log_2_FC | ≥ 1” and “*p* ≤ 0.05 and | FC | ≥ 1.2 or FC < 0.83” as the threshold to select subsets of DEGs and DAPs, respectively. DEGs encoding protein sequences were compared to the nucleotide sequences using Blastp as follows: (i) E-value less than 1e-15, (ii) the results of the highest score. The correlation coefficient was calculated between DEGs and DAPs by Spearman’s correlation test.

### Bioinformatic analysis of DEGs

Bioinformatics mainly includes GO function annotation and KEGG metabolic pathway enrichment analysis. GO annotation includes three levels: biological process (BP), cell component (CC), and molecular function (MF). GO function analysis of DEGs/DAPs can explain the enrichment of differential genes or protein and the differences between samples at the gene function level. KEGG is a public database, which uses molecular-level information to understand the function and utility of biological systems through large-scale molecular datasets generated by genome sequencing and other high-throughput experimental technologies.

### Quantitative real-time PCR analysis

In order to verify the reliability of transcriptome data, the quantitative real-time PCR (qRT-PCR) was performed by using the biological replication of RNA used for transcriptome sequencing. RNA samples were reverse transcribed into cDNA using the Takara gDNA quantitative reverse transcription kit. Twenty genes were randomly selected for qRT-PCR verification of RNA-seq results, qRT-PCR validation was performed using novozyme biological qPCR master mix, and the reaction procedure was as follows: 10 μl of 2 × aceq qPCR SYBR Green master mix, 0.4 μl of positive and negative primers, 2 μl of cDNA, and 7.2 μl of ddH_2_O. The reaction was carried out on a Bio-Rad CFX96TM Real-Time System according to the following procedure: pre-denaturation at 95°C for 30 s → cyclic reaction at 95°C for 10 s, 60°C for 30 s, 39 cycles → melt curve from 95°C for 15 s, 60°C for 60 s, and 95°C for 10 s. The relative expression of actin gene (three biological replicates) was calculated by the *Cq* value comparison method (2^−ΔΔCq^ method × 3 technical repetitions). Detailed information on the genes and primers in this experiment is listed in **Table 1**.

**Table 1:** Genes and primers used for the RNA-seq quantitative real-time PCR analysis.

No.	Gene ID	Annotation description (predicted)	Primers used for qRT-PCR	
			F (5′-3′)	R (5′-3′)
*g1*	*Glyma.11G155000*	Uncharacterized protein	ACTACGTACGTTGCTCCTTTTA	TCATTTTCACGCAACTACTTCG
*g2*	*Glyma.13G242300*	Polyphenol oxidase	TTCAAGTTCCCTCCTTCTAACC	GTTGGGTGAAATTACGTGGATC
*g3*	*Glyma.15G024600*	Beta-fructofuranosidase, cell wall isozyme	GAAAGCTTACGTGTGAATCCTG	ACGAGAAGCAAAAACTAGCAAG
*g4*	*Glyma.17G030100*	Nodulin-13	CTTCTTGTCGTGTGTTGCTTTA	ATGATGGGTACACAAACCAGTT
*g5*	*Glyma.20G159400*	Uncharacterized protein	GAGGAATGGCTGAACTGAAAAG	GTTAATCGGAGGAGTAGCTGTG
*g6*	*Glyma.05G123600*	WRKY35	CACTCAAGCCTTCTAGTATGGT	TGTCCACCAAGCAATGATCTAT
*g7*	*Glyma.05G184500*	WRKY51	GCTGTGTCAATGCATTGTCTAA	AACAAGACGAAGCATTCCATTC
*g8*	*Glyma.09G280200*	WRKY33	ACATCAAAGCTGTGATCACAAC	GCTGATATGTTGCTGTTCTGAG
*g9*	*Glyma.18G213200*	WRKY70	CACTTTTGATAGCTCACCAAGG	CATCATCAAGCTGAGCAGAATC
*g10*	*Glyma.18g074100*	HSP80	GAATGTTGAAGCTTGGACTGAG	AAAAACATAGCCAACTCCGTTC
*g11*	*Glyma.17G030200*	Stress-induced protein	AGAGCCAGTTGATGTCTTTGTA	CGTACTAATGCATTTGCCTCAA
*g12*	*Glyma.15G199700*	Uncharacterized protein	TCATTGATGTAGAACGGGGAAA	CACCGTCCATTTTACATTTCGT
*g13*	*Glyma.14G139600*	Proteasome non-ATPase regulatory subunit homolog	CGAAATTGCTTTGCGAGATTTC	GGTTCAATCAATCTGCAAAGGT
*g14*	*Glyma.12G218200*	MYB51	ATCATCAGCTATGCAACGTTTC	CCCTCGTTGTTGTTACACAAAT
*g15*	*Glyma.10G184700*	Inhibitor of trypsin and Hageman factor	TGATAGGGTTTGGGTTTGGATC	CTAGTGGGGAGCAGTTTCTATC
*g16*	*Glyma.08G332900*	HSP 80	TAGATTGGTGTAACGTGAACCA	AGGAAGAAAATGGGAATAGGCA
*g17*	*Glyma.08G041500*	Protein CUP-SHAPED COTYLEDON	TAAATCTGAACCTTGGGACCTC	TGCCCATATAAAAGACCAAGGT
*g18*	*Glyma.04G230000*	Uncharacterized protein	TCTTCATTTGGTGCTCTTTTGG	TTGTTGTAGTAATTTTCGCCCG
*g19*	*Glyma.07G154700*	Uncharacterized protein	GAGACTGGAGGCATCATTCATA	CTGTGAGTTGGAACTATTGCAC
*g20*	*Glyma.15G251500*	Probable glutathione S-transferase	GGAGTTCATAGAGGCCCTTAAA	AAAAGTCTTGAACCAAGTGTCG
*g21*	*Glyma.12G234800*	Alpha-amylase/subtilisin inhibitor	CTCCCAGTTGTGTTTGAGAAAG	GCTCCAATCATTTCACAACTGT
*g22*	*Glyma.20G205800*	Griseus protease inhibitor	CATCGTCACCGCTGATTTTC	TTCTTGGAGCCTGATAAACAATTCC
*g23*	*Glyma.07G200700*	HSP17.5	GCTTCTATGGAAAATGGGGTTC	ATCCTCAGCAAAAGAGAACAGA
*g24*	*Glyma.14G099900*	HSP17.9	TGCTTGAACAACTTGTAGAACG	TGGACAGCACAGAGTAACTTAG
*g25*	*Glyma.19G011400*	HSP22.7	TTTGTTTGGGACATGGGATTTC	GATCAACAAAAGAAGCGTCACT
*g26*	*Glyma.09G131500*	HSP 90-1	GCCAAAGGTGTTGTGTTAATGA	GCGCATTGACTACATTCCTTAG
*g27*	*Glyma.08G106700*	Peptidyl-prolyl cis-trans isomerase	AGGAGAAACAGAAGACTAAGGC	TCAAGCTCTAAAACCTTTGTGC
*g28*	*Glyma.16G206200*	HSP22.0	AGACAATGTGGACTTGGATTCT	CAGGTGACAACTTGTTCATTGT
*g29*	*Glyma.09G042200*	Pectinesterase	GCAATGGCTTTTGGTTTAAACC	GAACTGAGTTTCGATGAGCTTC

## Results

### The effect of HT treatment on the physiological indexes of JD21 and HD14

**Figure 1:**
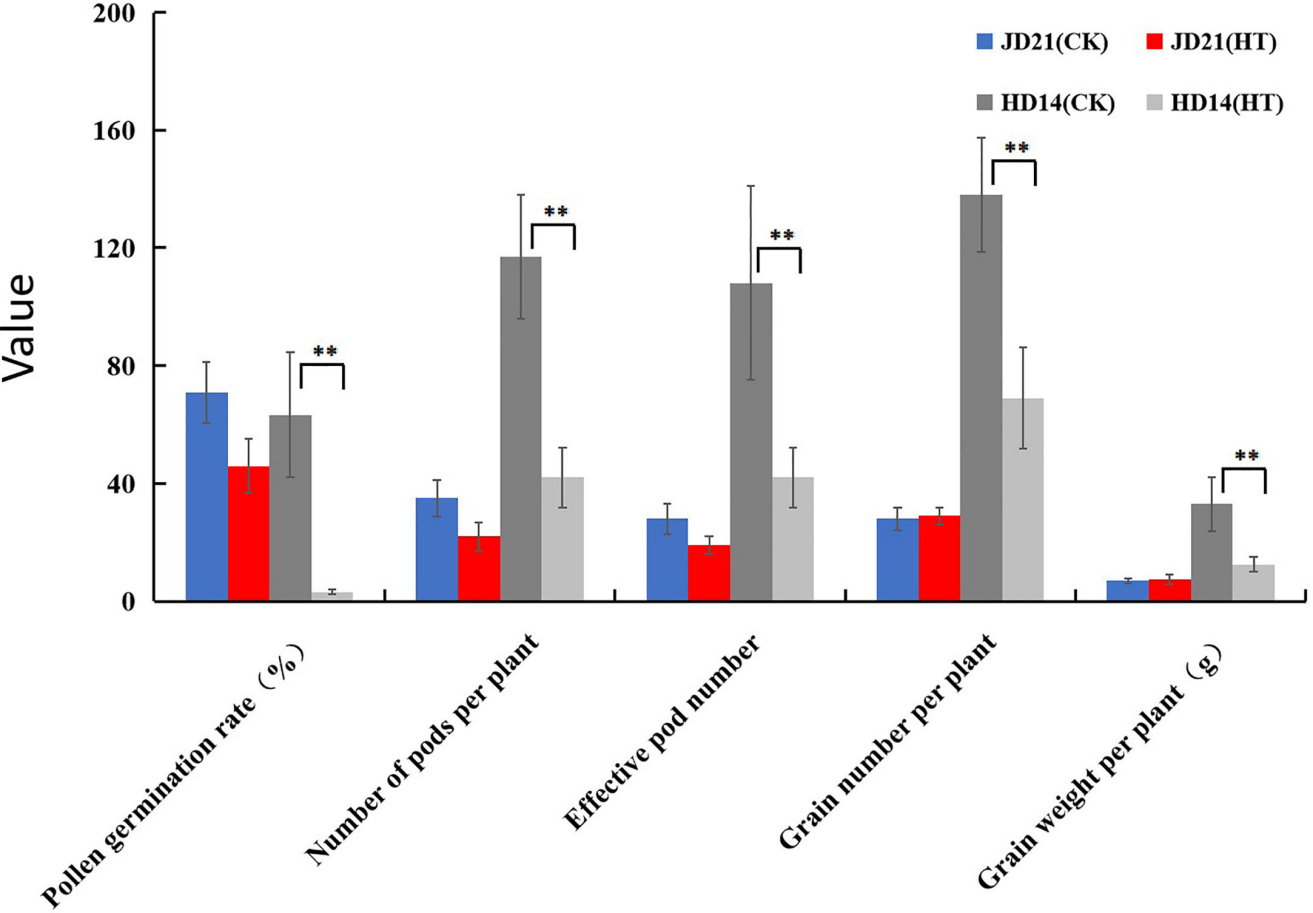
Determination analysis of physiological indexes of JD21 and HD14 under HT stress. **represents a significantly difference (P < 0.01).

Soybean JD21 and HD14 plants were artificially warmed by covering with plastic film at 09:00 each day (for a continuous period of 7 days) at the beginning of the flowering stage. The temperature was recorded throughout the experimental period. The continuous HT treatment of soybean plants resulted in a significantly higher temperature (43.40 ± 1.70°C) compared with the ambient temperature in the field (36.85 ± 1.32°C). Under the condition of HT stress, the cell tissue structure and membrane stability of sensitive soybean will be seriously damaged, while the HT-resistant varieties are less affected by HT. Pollen germination rate, pod number per plant, effective pods per plant, seeds per plant, and seed weight per plant reduced by 50.00%–94.99% of HD14 after HT stress. However, pollen germination rate, pod number per plant, and effective pods per plant of JD21 only decreased by 21.21%, 37.14%, and 32.14%, while seeds number per plant and seed weight per plant did not decrease after HT stress. Results showed that compared with the field environment, the changes of pollen germination rate, pod number per plant, effective pods per plant, seeds per plant, and seed weight per plant of JD21 after HT stress were less than those of HD14 (**Figure 1**).

### Analysis of differential transcriptome response to HT stress

#### Sequencing data quality preprocessing results

**Figure 2:**
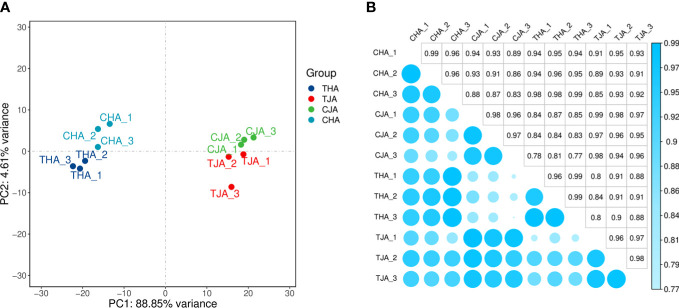
Quality analysis of the transcriptomics data. **(A)** PCA of the anther transcriptomes from the control and high-temperature-treated samples. **(B)** Heatmap coefficient matrix from the control and high-temperature-treated samples.

The anthers of soybean cultivars with JD21 and HD14 were sequenced by Illumina sequencer. The transcriptome sequences of 12 samples were analyzed and sequenced, and 180.54 G clean data was obtained. The effective data of each sample were distributed in 6.19–8.21 G, the Q30 base was distributed in 92.74%–95.83%, and the average GC content was 44.74%. By comparing the reads to the reference genome, the genome comparison of each sample obtained a response rate of 95.85%–97.95% (**Table 2**). Based on the comparison results, the expression of protein coding genes was analyzed. Principal component analysis (PCA) based on the transcriptome analysis exhibited an obvious separation among JD21 and HD14 pollen grains (**Figure 2A**). The correlation of protein coding gene expression levels among samples is an important indicator to test the reliability of experiments and the rationality of sample selection. In this study, the correlation coefficients between samples are high (**Figure 2B**), indicating that the similarity of expression patterns between samples is high, and the sequencing quality is reliable. All of these RNA-Seq reads were deposited in the Sequence Read Archive Database (http://www.ncbi.nlm.nih.gov/Traces/sra/) under the accession number PRJNA877709 in this study.

**Table 2:** Number of reads sequenced and mapped to the soybean genome.

Sample	Raw_reads	Raw_bases	Clean_reads	Clean_bases	Valid_bases	Q30		GC
CHA_1	58.84M	8.83G	56.37M	8.16G	92.43%	93.95%		44.48%
CHA_2	55.34M	8.30G	54.04M	7.76G	93.53%	95.83%		44.55%
CHA_3	50.46M	7.57G	48.98M	6.93G	91.57%	95.42%		44.52%
CJA_1	49.92M	7.49G	48.02M	6.88G	91.84%	94.18%		44.62%
CJA_2	45.13M	6.77G	41.83M	6.19G	91.39%	93.77%		44.93%
CJA_3	50.08M	7.51G	48.02M	6.91G	91.96%	93.88%		44.68%
THA_1	54.49M	8.17G	52.49M	7.49G	91.63%	94.40%		44.79%
THA_2	58.86M	8.83G	56.96M	8.05G	91.14%	94.79%		44.50%
THA_3	56.93M	8.54G	54.98M	7.79G	91.25%	94.60%		44.70%
TJA_1	57.65M	8.65G	53.82M	7.38G	85.37%	92.74%		44.77%
TJA_2	57.95M	8.69G	55.76M	7.88G	90.59%	94.24%		44.79%
TJA_3	53.69M	8.05G	51.94M	7.38G	91.66%	94.65%		44.60%
Sum	649.34M	97.4G	623.21M	88.8G	91.20%	94.37%		44.66%

#### Identification of DEGs by RNA-Seq

**Figure 3:**
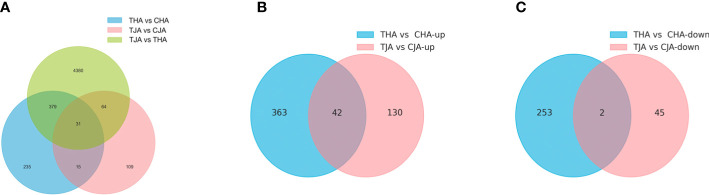
Venn diagram of differentially expressed genes (DEGs) in anthers of two soybean cultivars in response to high-temperature (HT) treatment. TJA, THA represents anther of JD21 and HD14 after HT stress; CJA, CHA represents anther of JD21 and HD14 in a normal environment. **(A)** Venn plot of DEGs of THA vs. CHA, TJA vs. CJA, and TJA vs. THA. **(B)** The number of upregulated genes between (HT) treatment between in THA vs. CHA and TJA vs. CJA by using a Venn diagram. **(C)** The number of downregulated genes between (HT) treatment between in THA vs. CHA and TJA vs. CJA by using a Venn diagram.

A total of 46,941, 47,147, 47,600, and 47, 085 genes in TJA vs. CJA, THA vs. CHA, TJA vs. THA, and CJA vs. CHA were obtained, respectively, matching the soybean reference genome. Then, “*p* < 0.05 and |Log_2_FC|≥1” were used as the threshold to screen the DEGs in the three comparison groups. It was found that there were 219, 660, 4,854, and 3,959 DEGs between TJA vs. CJA, THA vs. CHA, TJA vs. THA, and CJA vs. CHA, respectively (**Table S1**), among which, 172 upregulated and 47 downregulated in TJA vs. CJA, 405 upregulated and 255 downregulated in THA vs. CHA, 2,662 upregulated and 2,192 downregulated in TJA vs. THA, and 2,090 upregulated and 1,869 downregulated in CJA vs. CHA (**Figure 3**, **Figure S1**). JD21 and HD14 shared 46 DEGs in total, and more specific DEGs (660) were found in sensitive genotypes (THA vs. CHA) under HT stress, while only 219 were found in tolerant genotypes (TJA vs. CJA) (**Figure 3**). These results suggest that different HT-tolerant varieties have different mechanisms in response to HT stress. Compared to the HT-sensitive variety (HD14), there were more DEGs upregulated in the HT-tolerant variety (JD21); this may be the reason why JD21 is more HT-resistant than HD14.

In the TJA vs. CJA comparison group, six extreme DEGs with a multiple difference of at least 20 times are identified, mainly including *Glyma.11G155000*, *Glyma.13G242300*, *Glyma.15G024600*, *Glyma.17G030100*, *Glyma.17G030200*, and *Glyma.20G159400*. Compared with the control (CJA), the high expression of these DEGs in JD21 anthers after HT treatment may endow it with HT tolerance. In addition, a number of DEGs related to response to HT and flower development were also found in different comparisons, mainly including transcription factors (such as WRKY translation factor, GATA translation factor, heat stress translation factor, and MYB translation factor), heat shock proteins (HSPs), glutathione S-transfer, and ATP synthesis. These DEGs may be key candidate genes for soybean anther response to HT stress.

### Bioinformatic analysis of DEGs

#### GO annotation analysis of DEGs

**Figure 4:**
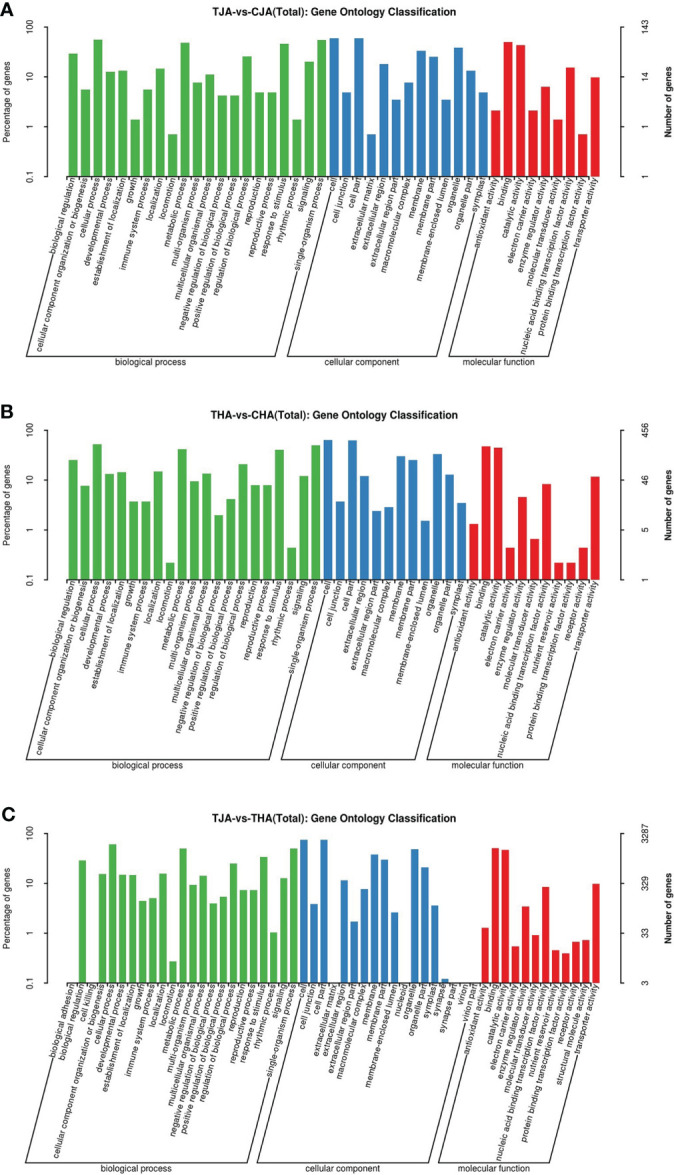
Gene ontology (GO) annotation terms of DEGs between TJA and CJA, THA and CHA, and TJA and THA. TJA, THA: anthers of JD21 and HD14 after high-temperature (HT) treatment; CJA, CHA: anthers of JD21 and HD14 in the control environment. **(A)** Significant (*p* < 0.01) GO terms for DEGs between TJA and CJA. **(B)** Significant (*p* < 0.01) GO terms for DEGs between THA and CHA. **(C)** Significant (*p* < 0.01) GO terms for DEGs between TJA and THA.

To understand the functions of DEGs, these genes were divided into three categories for analysis: biological process (BP), cellular component (CC), and molecular function (MF). One hundred forty-three (65.3%) from the TJA vs. CJA comparison group were annotated into 43 functional categories, namely, 21 BP, 13 CC, and 9 MF. Four hundred fifty-six (69.1%) from the THA vs. CHA comparison group were annotated into 32 functional categories, namely, 12 BP, 11 CC, and 9 MF. Three thousand two hundred eighty-seven (67.7%) from the TJA vs. THA comparison group were annotated into 53 functional categories, namely, 23 BP, 18 CC, and 12 MF. DEGs of the three groups were significantly enriched in nine molecular functions: cellular process, metallic process, response to stimulus, single organization process, cell, membrane, organelle, binding, and catalytic activity (**Table S2**; **Figure 4**). These GO terms are mainly involved in defense response, response to biological stimuli, auxin-activated signaling pathway, and other biological processes. Since these pathways are enriched with a large number of DEGs, it indicates that these pathways are involved in the soybean response to HT stress.

#### KEGG pathway enrichment of DEGs

**Figure 5:**
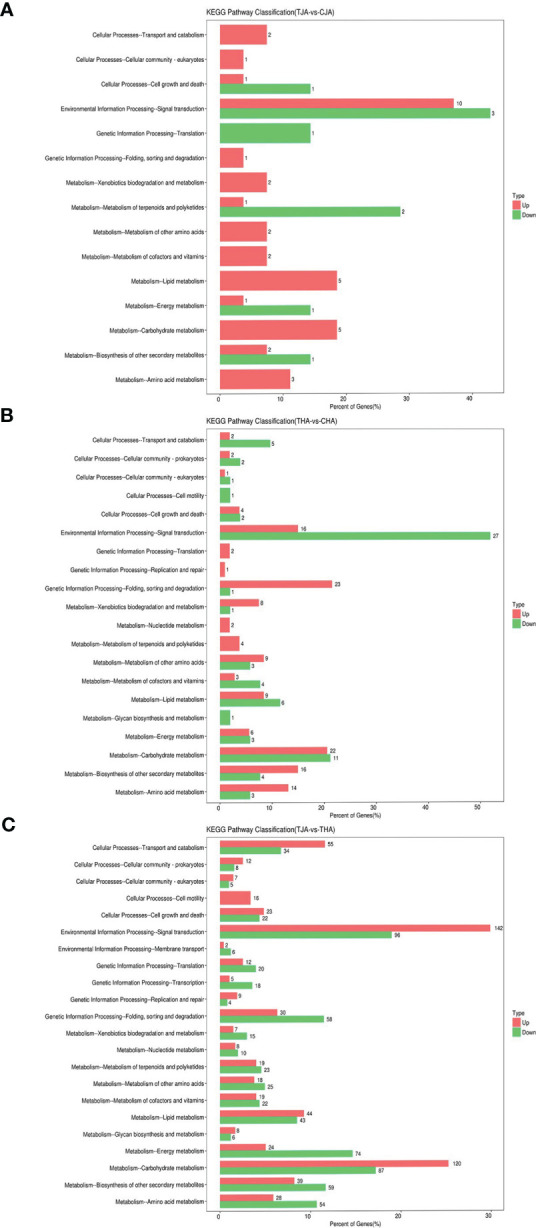
KEGG pathways significantly enriched of DEGs between TJA and CJA, THA and CHA, and TJA and THA. TJA, THA: anthers of JD21 and HD14 after high-temperature (HT) treatment; CJA, CHA: anthers of JD21 and HD14 in the control environment. **(A)** Significant enriched KEGG pathways for DEGs between TJA and CJA. **(B)** Significant enriched KEGG pathways for DEGs between THA and CHA. **(C)** Significant enriched KEGG pathways for DEGs between TJA and THA.

To investigate the involvement of DEGs in biological functions, 219, 660, and 4,854 DEGs in comparisons between TJA vs. CJA, THA vs. CHA, and THA vs. TJA were mapped to 52, 94, and 190 pathways in the KEGG database (**Table S3**). Among these significantly enriched pathways, the common metabolic and the most represented pathways were “Plant hormone signal transduction”, “MAPK signaling pathway-plant”, “Starch and sucrose metabolism”, “Glutathione metabolism”, and Phenylpropanoid biosynthesis”. THA vs. CHA enriched more DEGs than TJA vs. CJA (**Figure 5**), indicating that HD14 was more sensitive to HT stress compared to JD21. This is also consistent with the previous HT treatment effect and physiological response of JD21 and HD14 results.

### Combined analysis of differential transcriptome and proteome results

#### Analysis of common DEGs/DAPs

In the previous research results (Li et al., 2020, iTRAQ data can be found online at https://doi.org/10.1016/j.jprot.2020.103968), the proteins with fold change > 1.2 (*p* < 0.05) were upregulated between the control group and HT treatment, and the proteins with fold change < 0.83 (*p* < 0.05) were downregulated. A total of 479, 371, and 417 DAPs were determined in the comparison of TJA vs. CJA, THA vs. CHA, and TJA vs. THA, and the DAPs of this result were jointly analyzed with the DEGs of the RNA-seq results above; there are 2, 26, and 67 common DEGs/DAPs in TJA vs. CJA, THA vs. CHA, and TJA vs. THA, respectively (**Table 3**, **Table S4**). The protein and gene information with the same expression pattern can further explain organisms and some important mechanisms and help to find important markers. There were 1, 24, and 54 common DEGs/DAPs that have the same expression pattern between TJA vs. CJA, THA vs. CHA, and TJA vs. THA at the protein and gene level, respectively, of which 1, 23, and 41 common DEGs/DAPs are upregulated and 0, 1, and 13 common DEGs/DAPs are downregulated. The protein and gene information with opposite expression patterns not only enriches the available data of biological systems, but also explains the phenomenon that cannot be explained by a single technology. In TJA vs. CJA, THA vs. CHA, and TJA vs. THA, there are 1, 2, and 13 common DEGs/DAPs with opposite expression patterns at the protein level and gene level, respectively.

**Table 3:** The correlation number relationship between genes and proteins in the significant differences range.

Group name	Number of proteins	Number of genes	Number of correlations
THA vs. CHA	371	660	26
TJA vs. CJA	479	219	2
TJA vs. THA	417	4,854	67

#### Expression pattern analysis of common DEGs/DAPs

Proteome and transcriptome reflect gene expression from two different levels. One of the purposes of joint analysis is to achieve data complementarity and obtain more complete expression information of organisms. Based on the change patterns at the gene and protein levels, the expression correlation between genes and proteins was divided into four groups.

##### Group I: the expression patterns of genes and proteins are the same

The protein and gene information with the same expression pattern can further confirm and explain some important mechanisms of organisms and help to find meaningful markers. The sequencing results of protein level and gene level were analyzed. There was 1 common up DEGs/DAPs (glutathione S-transferase) in TJA vs. CJA and the same expression pattern at the gene level and protein level, but downregulated in DEGs/DAPs was not found. Twenty-four common DEGs/DAPs in THA vs. CHA changed at the gene level and protein level, and the change pattern was basically the same, of which 23 DEGs/DAPs were upregulated and 1 DEGs/DAPs was downregulated; these common up DEGs/DAPs mainly include 14 uncharacterized proteins, 5 HSPs, 2 peptidylprolyl isomerase, 1 heat shock transcription factor, and 1 Tau class glutathione S-transferase. Fifty-four common DEGs/DAPs in TJA vs. THA had the same expression pattern at the gene level and protein level, of which 41 DEGs/DAPs were upregulated and 13 DEGs/DAPs were downregulated. These common up DEGs/DAPs mainly include S-adenosylmethionine synthase, peptidyl-prolyl cis-trans isomerase, beta-galactosidase, lipoxygenase, profilin, peroxidase, and uncharacterized protein. Compared with THA, the high expression (41 up and 13 down) of these DEGs/DAPs in TJA may be one of the reasons why JD21 is more resistant to HT than HD14.

##### Group II: the expression pattern of gene is opposite to that of protein

Protein and transcriptome reflect gene expression from two different levels. Another purpose of joint analysis is to compare the differences between data. The protein and gene information with opposite expression patterns not only enriches the available data of biological systems, but also explains the phenomena that cannot be explained by a single technology. In this study, there were 1 common DEGs/DAPs (Uncharacterized protein) with opposite expression patterns in TJA vs. CJA, 2 common DEGs/DAPs (Uncharacterized protein) with opposite expression patterns in THA vs. CHA, and 13 common DEGs/DAPs with opposite expression patterns in TJA vs. THA. This protein and gene information with opposite expression patterns not only enriches the available data of biological systems, but also explains the phenomena that cannot be explained by a single technology.

##### Group III: the protein remains unchanged, but the gene level significantly changed

Based on the same method, it was analyzed that the expression patterns of protein level remained unchanged with the gene level significantly changed instead. The results showed that only one gene changed significantly (upregulated) at the gene level in TJA vs. CJA, but remained unchanged at the protein level. Eight DEGs changed significantly at the gene level in THA vs. CHA, but remained unchanged at the protein level. Among the 8 DEGs with significant changes at the transcriptional level, 6 were upregulated and 2 were downregulated, and among the 109 DEGs with significant changes in TJA vs. THA, 41 were upregulated and 68 were downregulated.

##### Group IV: the gene level remains unchanged, while the protein level significantly changed

Similarly to the above, 403 DAPs showed significant changes at the protein level in TJA vs. CJA, while the gene level remained unchanged, of which 158 were upregulated and 245 were downregulated. There were 305 DAPs that expressed significant changes at the protein level, while the gene level remained unchanged in THA vs. CHA. Of the 305 DAPs with significant changes in protein level, 85 were upregulated and 220 were downregulated; 283 DAPs showed significant changes at the protein level in TJA vs. THA, while the gene level remained unchanged, of which 181 were upregulated and 102 were downregulated.

#### Bioinformatics analysis of common DEGs/DAPs

Due to the incompleteness and complementarity of transcriptome and proteome research methods, previous studies generally tend to integrate these two results, obtaining a “panorama” of expression change response to HT stress and realizing the complementarity and integration of data. Therefore, the focus of the next research is to analyze genes/proteins and their regulatory networks with the same or opposite expression patterns at the gene and protein levels.

#### GO annotation analysis of common DEGs/DAPs

##### GO annotation analysis of DEGs/DAPs with the same expression pattern at gene and protein levels

The results of GO analysis based on sequence homology showed that two common DEGs/DAPs with the same expression pattern in TJA vs. CJA were annotated into 11 GO functional groups, namely, 4 BP, 2 CC, and 5 MF. Each GO function was enriched to only 1 DEGs/DAPs. Twenty-four common DEGs/DAPs with the same expression pattern in THA vs. CHA were annotated into 39 GO functional groups, namely, 19 BP, 9 CC, and 11 MF. In the BP category, the most abundant DEGs/DAPs are chaperone-mediated protein folding and response to heat; in the CC category, the most abundant DEGs/DAPs are cytoplast and nucleus; in the ML category, the most abundant DEGs/DAPs is ATP binding. In TJA vs. THA, 54 common DEGs/DAPs with the same expression pattern were annotated into 148 GO functional groups, namely, 57 BP, 30 CC, and 61 MF. In the BP category, the most abundant DEGs/DAPs are cell wall organization and carbohydrate metallic processes. In the CC category, the most abundant DEGs/DAPs are extracellular region, nucleus cell wall, and cytoplast. Among the MF categories, the most abundant DEGs/DAPs are metal ion binding, polygalacturonase activity, specific esterase activity, aspartyl esterase activity, and ATP binding (**Table S5**).

##### GO annotation analysis of DEGs/DAPs with opposite expression patterns at gene and protein levels

Compared with DEGs/DAPs with the same gene and protein expression patterns, DEGs/DAPs with opposite gene and protein expression patterns were much less, and the annotated GO is also relatively reduced. One common DEGs/DAPs with opposite expression pattern in TJA vs. CJA was annotated into nine GO functional groups, namely, three BP, two CC, and four MF. In THA vs. CHA, two common DEGs/DAPs with opposite expression patterns are annotated into three GO functional groups, namely, one BP and two MF, but CC is not annotated. Thirteen common DEGs/DAPs with opposite expression patterns in TJA vs. THA were annotated into 44 GO functional groups, namely, 22 BP, 10 CC, and 12 MF (**Table S6**).

#### KEGG annotation analysis of common DEGs/DAPs

##### KEGG annotation analysis of DEGs/DAPs with the same expression pattern at gene and protein levels

KEGG pathway enrichment analysis showed that one common DEGs/DAPs with the same expression pattern in TJA vs. CJA is enriched into four metabolic pathways, and each pathway is enriched into one DEGs/DAPs. The four pathways are glutathione metabolism, metabolism of xenobiology by cytochrome P450, drug metabolism—cytochrome P450, and drug metabolism—other enzymes. Among 24 common DEGs/DAPs with the same expression pattern in THA vs. CHA, 20 DEGs/DAPs were enriched into 9 metabolic pathways, among which, 17 DEGs/DAPs enriched by protein processing in endoplasmic reticulum, and 2 DEGs/DAPs enriched by PI3K Akt signaling pathway and necroptosis. Among the 54 common DEGs/DAPs with the same expression pattern in TJA vs. THA, 28 DEGs/DAPs were enriched into 30 metabolic pathways, and the most enriched pathways were pentose and gluconate interconversion, protein processing in endoplasmic reticulum, regulation of actin cytoskeleton, and phenopropioid biosynthesis, which were enriched into 8, 6, 4, 3, and 13 DEGs/DAPs, respectively (**Table S7**). Among the pathways in all groups, four pathways appear in each group: glutathione metabolism, metabolism of xenobiology by cytochrome P450, drug metabolism—cytochrome P450, and drug metabolism—other enzymes.

As the research objectives of these omics studies are all within the same network, there is inevitably a certain correlation between them. In this study, the transcriptomic and proteomic data were further integrated in order to identify key genes involved in soybean’s tolerance to HT stress. Whether transcriptome-focused or combined proteome-focused KEGG analysis, both enriched Cytochrome P450 (CYP), which is a group of multifunctional enzymes that plays an important role in xenobiotic metabolism; Glutathione metabolism, which serves as an electron donor for many enzymes; and MAPK (mitogen-activated protein kinase) signaling pathway, which is an important transmitter of signals from the cell surface to the nucleus. This suggests that high-temperature stress most likely affected the metabolism of these substances.

### qRT-PCR validation

**Figure 6:**
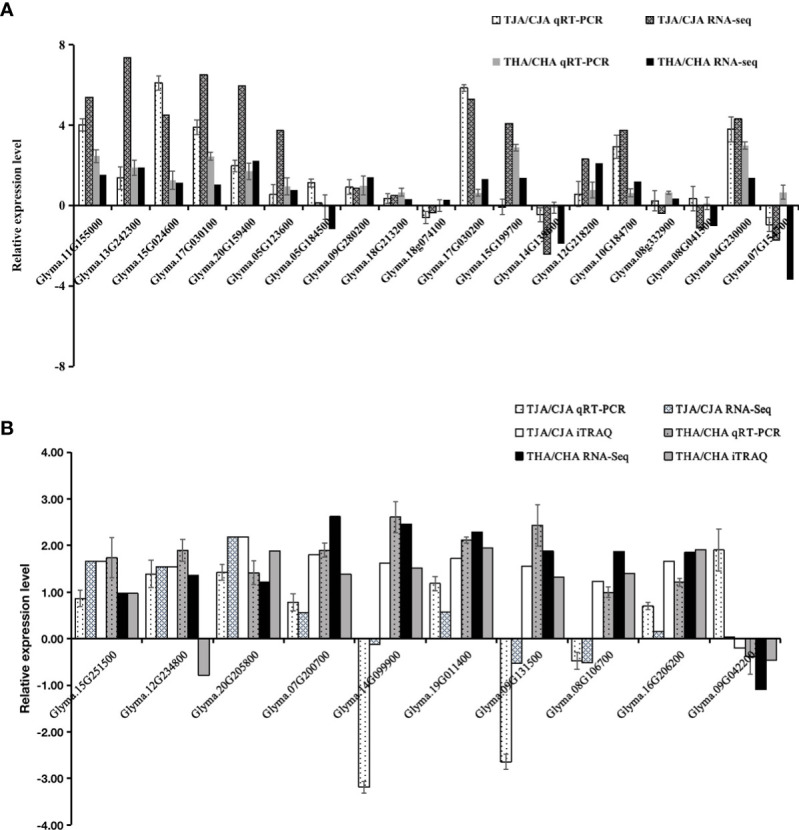
qRT-PCR analysis. **(A)** Comparison between qRT-PCR and RNA-seq results for differentially expressed genes (DEGs). **(B)** Comparison between qRT-PCR/iTRAQ and RNA-seq results for differentially abundant genes (DEGs)/proteins (DAPs).

To verify the reliability of RNA-seq results, 19 DEGs from RNA-seq results were randomly selected for qRT-PCR verification, and the samples used in qRT-PCR were in the same manner as the RNA-seq and iTRAQ samples. The expression pattern of 16 (84%) of the verified 19 DEGs was similar to that of TJA vs. CJA RNA-seq, indicating that the results of RNA-seq were reliable (**Figure 6A**). At the same time, 10 common DEGs/DAPs from joint analysis results were randomly selected for verification. The results indicated that 10 genes showed the same trend (100%) between RNA-seq and qRT-PCR, and the expression trend of 9 genes was the same (90%) between iTRAQ and qRT-PCR in the THA vs. CHA group. In the TJA vs. CJA group, qRT-PCR detected that nine genes’ (90%) expression patterns were consistent with RNA-seq, and six genes’ (60%) expression patterns were consistent with iTRAQ (**Figure 6B**). Coincidence analysis between RNA-Seq, iTRAQ, and qPCR results showed consistency, indicating the reliability of sequencing expression profile (gene and protein) in this study.

## Discussion

**Figure 7:**
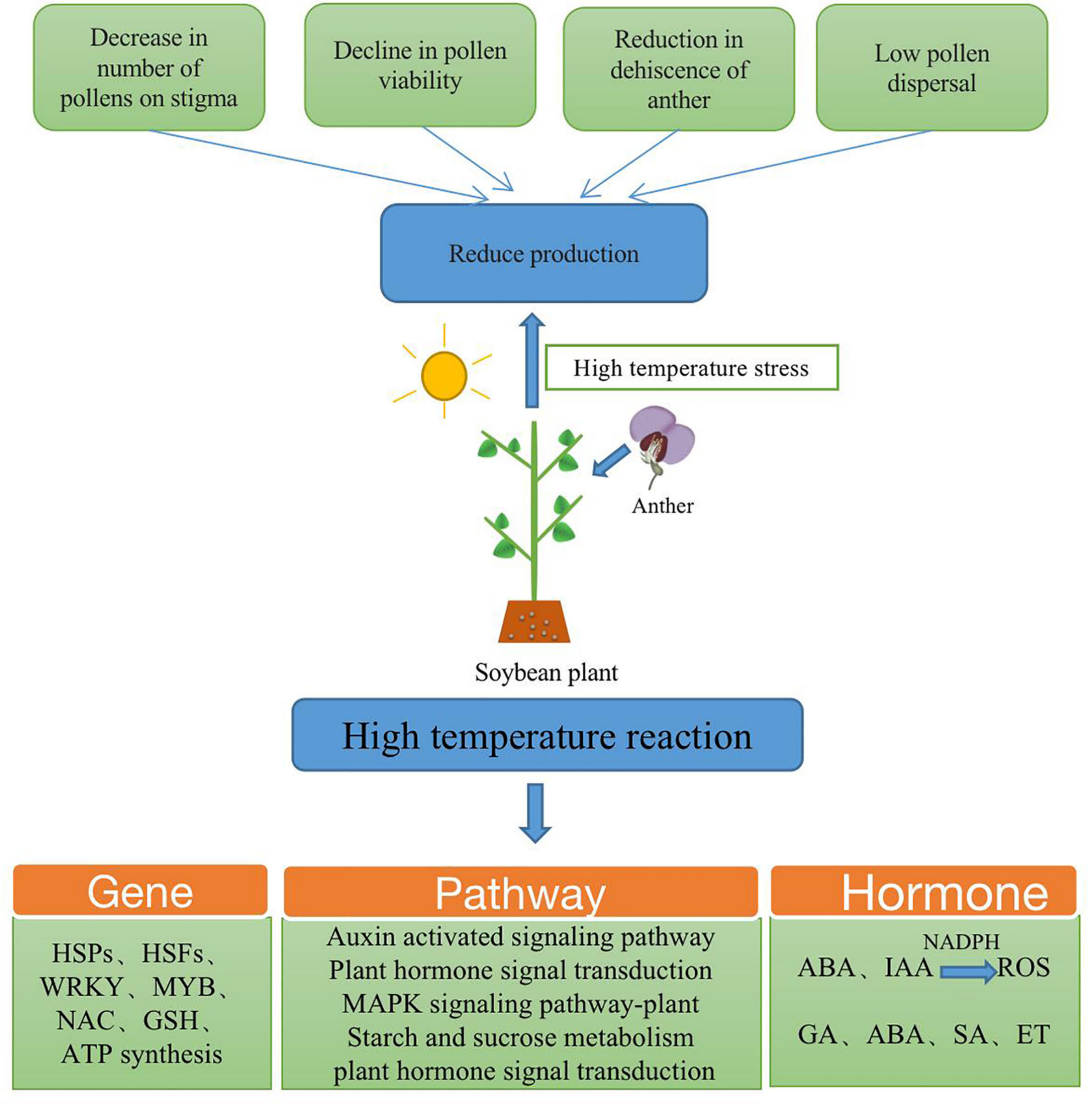
Response mechanism of soybean anthers to high-temperature stress.

Temperature plays a key environmental role that regulates the growth and development of plants. It has become one of the important factors that restrict its yield and quality, with high temperature affecting its normal growth. Existing research shows that HT stress leads to an increase in SOD enzyme activity, the relative conductivity of leaves, a decrease in relative water content, a significant decrease in seed germination rate and activity, and, finally, yield reduction (Ren et al., 1999; Han et al., 2001). To understand the regulation mechanism of soybean response to HT stress, the anthers of HT-resistant (JD21) and HT-sensitive (HD14) varieties treated with HT stress and control were analyzed by transcriptomics and proteomics to determine the biological processes and metabolic changes to adapt soybean, mining the key genes and metabolic pathways of flowering (anther) response to HT stress in soybean (**Figure 7**).

To understand the effect of HT stress on gene expression pattern, the transcriptome results were analyzed. In TJA vs. CJA, THA vs. CHA, and TJA vs. THA groups, 172, 405, and 2,662 DEGs were upregulated while 47, 255, and 2,192 DEGs were downregulated respectively, indicating that most genes were upregulated and induced by HT stress. At the same time, the RNA-seq results were correlated with the previous iTRAQ results (Li et al., 2020). There were 1, 26, and 67 common DEGs/DAPs in TJA vs. CJA, THA vs. CHA, and TJA vs. THA, respectively. These results indicated that soybean anther responds to HT stress by increasing the expression of these genes. It has been reported that after 6 h and 4°C low-temperature stress, low-temperature sensitive varieties have more DEGs than low-temperature resistant varieties, and low-temperature resistant varieties have upregulated DEGS (586), which was much higher than the downregulated DEGs (478), while the downregulated DEGs (133,5) in low-temperature sensitive varieties were much higher than the upregulated DEGs (243), indicating that the regulation mechanisms of HT and low temperature varied a lot (Pradhan et al., 2019). In this study, there were 441 DEGs in the THA vs. CHA group (HT-sensitive varieties) compared with the TJA vs. CJA group (HT-tolerant varieties), indicating that HT-sensitive varieties are easily affected by HT stress and change the expression mode of more genes.

### Heat shock proteins’ response to HT stress and flower development

HSPs are closely related to plant response to HT stress and are the main participants in plant resistance to HT. HT stress stimulates the upregulated expression of heat shock genes. These genes encode HSPs and play an important role under stress conditions (Muhammad et al., 2018). Studies have confirmed that HSPs, hormone regulation, ROS, and other metabolic pathways in plants are adaptive mechanisms in response to HT stress (Rizhsky et al., 2002; Larkindale and Vierling, 2008). The small HSPs family of genes is a major component of the 50 most highly induced genes in each heat stress treatment and is involved in the ROS pathway of the rapid response to heat stress, which also provides a basis for understanding the mechanisms of heat stress in plants (Zhou et al., 2022). It was found that HS/A9 can regulate the expression of a special heat shock transcription factor HSP during *Arabidopsis* seed development, thus mediating its response to HT stress. In addition, proteomic analysis of soybean leaves under HT and dry early interaction stress showed that proteins related to EF-Tu and HSPs were significantly upregulated under HT stress, which may be one of the main reasons for the enhancement of HT tolerance in soybean (Das et al., 2016), and it can be seen that HSPs play a key role in mediating plant response to HT stress. In this study, we found that many HSPs participate in the response to HT stress, such as *HSP90-1*, *HSP23.9*, and *HSP15.7*; these results showed that soybean plants could resist the adverse effects of HT stress by activating HSP family proteins.

### Transcription factors’ response to HT stress and flower development

Transcription factors are the major direction of plant stress research. It has been reported that many transcription factor families are closely related to the stress and flower development. WRKY family is a unique family of transcription factors in plants, which has been proved to play an important role in response to biological, abiotic stress and flower development. *WRKY10* overexpression promotes the accumulation of ROS in chloroplasts and plastid exosomes and induces the expression of heat-stimulated transcription factors and protein genes (Chen et al., 2022). *CaWRKY27b* was phosphorylated by *CaCDPK29* in a Ca^2+^-dependent manner and translocated from the cytoplasm to the nucleus to achieve transcriptional activation of *CaWRKY40*, which, in turn, positively regulated the tolerance of pepper to HT and humidity and resistance to cyanobacteria (Yang et al., 2022). In addition, *AtWRKY39* endows *Arabidopsis* with HT tolerance by regulating the signal pathways activated by SA and JA, while *AtWRKY*30 promotes heat tolerance of wheat by inducing gas exchange characteristics, antioxidant mechanism, biosynthesis of osmotic cells, and expression of stress-related genes (El-Esawi et al., 2019). Research showed that *AtWRKY25*, *AtWRKY26*, and *AtWRKY33* play positive roles in the synergy between ethylene activating protein and HSP and mediate the response of *Arabidopsis* to heat stress (Li et al., 2011). In this study, there were 2, 4, and 24 enriched significantly different WRKY genes in TJA vs. CJA, THA vs. CHA, and TJA vs. THA groups and joint analysis results, respectively. These upregulated or downregulated DEGs might help improve HT tolerance of soybean. In addition, MYB-like genes have been reported to transmit signals and regulate the expression of a variety of genes in response to heat stress (Hou et al., 2020). Zhao et al. (2017b) identified six HT tolerance genes from wheat through transcriptome sequencing, and further research showed that *TaMYB80* improved heat and drought tolerance of transgenic *Arabidopsis*, and ectopic expression of *Arabidopsis TaMYB68* in rape improved heat tolerance of rape. It was proved in multiple seasonal experiments at multiple locations that compared with non-transgenic *Arabidopsis*, the HT tolerance of transgenic *TaMYB* rape was improved (Deng et al., 2020). In this study, MYB-like DEGs were enriched in each group, and there were 4, 15, and 38 MYB-like DEGs in the TJA vs. CJA, THA vs. CHA, and TJA vs. THA groups, respectively. The *Glyma.08g059900* (MYB-like 102) was upregulated 16 times in TJA vs. CJA after HT stress, indicating that the gene is strongly responsive to the regulation of HT stress, and it can be used as a candidate gene for anther response to HT stress in soybean. Moreover, NAC transcription factors are also enriched in this study. Rice *OsNAC127* and *OsNAC129* can form heterodimers during rice filling and coordinate multiple ways to regulate seed development and heat stress response in rice reproductive stage (Ren et al., 2021). The activities of SOD, pod, and cat of rice *OsNAC006* mutant are reduced and the membrane lipid peroxidation index malondialdehyde (MDA) is reduced after HT and drought stress. The increased content indicates that *OsNAC006* plays a role in drought and heat tolerance by responding to stimulation, oxidoreductase activity, cofactor binding, and membrane-related pathways (Wang et al., 2020a), which indicates that the NAC family is involved in plant heat tolerance pathway.

### Effect of plant hormone signal transduction under HT stress

Plant hormones, including auxin (IAA), gibberellin (GA), abscisic acid (ABA), cytokinin (CK), salicylic acid (SA), ethylene (ET), and jasmonate (JA), play an important role in the process and signal network of plant response to stress (Bari and Jones, 2009). Studies have shown that HT stress can increase the level of ABA, and then ABA induces the expression of NADPH oxidase to induce ROS and enhances the antioxidant capacity by increasing the level of ROS, to improve the heat tolerance of plants (Suzuki et al., 2011). Studies have shown that exogenous application of IAA can effectively improve plant tolerance to HT and reduce the damage of HT stress to wheat anther development (Sakata and Higashitani, 2008), Kentucky bluegrass with more IAA and ABA has higher HT tolerance (Li et al., 2014a), and the heat stress effect of mustard seed and soybean stem after exogenous IAA treatment was effectively alleviated (Oberholster et al., 1991). Long-term HT stress of upland cotton would cause male sterility. Under HT stress, the target genes of mir160 of HT-sensitive upland cotton (H05) were inhibited by *ARF10* and *ARF17*, which activated the expression of IAA and enhanced the male sterility phenotype (Ding et al., 2017). In addition, compared with HT-resistant KSG1214, the accumulation of amino acids, ABA, and IAA complexes in anthers of HT-sensitive KSG1177 was significantly increased under HT stress, indicated that these hormones play an important regulatory role in enhancing wheat HT stress tolerance (Bheemanahalli et al., 2020). In this study, many DEGs are enriched to plant hormone signal transmission (ko04075), and most of these genes were auxin-induced protein and auxin-responsive protein, and there were 4 DEGs (2 upregulated and 2 downregulated) enriched in TJA vs. CJA, 28 DEGs (23 downregulated and 5 upregulated) enriched in THA vs. CHA, and 112 DEGs (39 upregulated and 73 downregulated) enriched in TJA vs. THA. To sum up, plant hormone signal transmission plays an important role in the process of plant stress tolerance.

### Effects of other metabolic pathways under HT stress

To adapt to abiotic stress, plants will change transcription, translation, protein level, enzyme activity, and plant hormone concentration to achieve homeostasis (Choi et al., 2000). MAPK (mitogen activated protein kinase) signaling pathway is an important transmitter of signal transmission from the cell surface to the nucleus. As a member of MAPK family, the expression of MPK increases under low temperature, high salt, and mechanical stress (Mizoguchi et al., 1996). *ZmMPK7* transgenic tobacco alleviates ROS-mediated plant osmotic stress injury (Zong et al., 2009). Chen et al. (2020) identified three MAPK *Arabidopsis* homologous genes *GhMAP3K14*, *GhMKK11*, and *GhMPK31* in cotton, and proved that they can regulate the tolerance of cotton to drought stress. Cytochrome P450 (CYP) is a group of multifunctional enzymes, which plays an important role in exogenous metabolism. Sorghum *CYP99A1* and *CYP709C1* are upregulated in cold-tolerant sorghum varieties, indicating that they regulate the tolerance to low-temperature stress (Chopra et al., 2015). Salt stress upregulates the expression of *TaCYP81D5*, and overexpression of this gene can accelerate ROS clearance and enhance salt tolerance in wheat seedlings and reproductive stages (Wang, 2020b). Glutathione (GSH) metabolism may play a crucial role in plant adaptation to the environment by acting as a nitric oxide (NO) reservoir (Foyer and Noctor, 2009; Malik et al., 2011). Glutathione metabolism is used as an electron donor for many enzymes, such as glutathione peroxidase (GPX), glutathione S-transferase (GST), and glutathione reductase (GR); GPX and GST improved the drought tolerance and low-temperature tolerance of *Euphorbia esula* (Anderson and Davis, 2004). The co-expression of tobacco GR resulted in the increased tolerance of transgenic plants to salt and low-temperature stress (Le et al., 2011). These metabolic pathways play an important role in the response of plants to abiotic stress, so they may also be involved in the regulation of HT stress, and their specific functions need to be further verified.

## Conclusions

In summary, we constructed a gene expression network in anther of HT-resistant (JD21) and HT-sensitive (HD14) varieties under HT stress and control, and some key candidate DEGs were identified in anther between different comparison groups’ response to HT stress by sequencing of transcriptome and proteome. Furthermore, the GO annotation and KEGG pathways of these genes were analyzed to explore the regulatory mechanism of soybean response to HT stress. Meanwhile, to better understand the molecular mechanism of soybean response to HT stress, the conjoint analysis of transcriptome sequencing and proteomics analysis are completed. Our results provided basic data to better understand the effect of HS on anther in soybean at a transcription and translation level and identified candidate genes that can be potentially used as targets for the subsequent molecular-assisted breeding of HT-resistant traits in soybean.

## Data availability statement

The datasets presented in this study can be found in online repositories. The names of the repository/repositories and accession number(s) can be found below: https://www.ncbi.nlm.nih.gov/, PRJNA877709.

## Author contributions

JL, XW, and LQ conceived and designed the contents. JL, LC, XZ, YL, MW, YS, ZT, HC, WL, LQ, and SZ conducted the experiments; JL, LC, JXW, and JTW wrote and revised the manuscript. All authors contributed to the article and approved the submitted version.

## Supplementary material

Supplementary Figure 1 The number of differentially expressed genes between TJA vs CJA, THA vs CHA, TJA vs THA and CJA vs CHA.

## References

[B1] Anderson J. V. Davis D. G. (2004). Abiotic stress alters transcript profiles and activity of glutathione s-transferase, glutathione peroxidase, and glutathione reductase in euphorbia esula. Physiologia plantarum 120 (3), 421–433. doi: 10.1111/j.0031-9317.2004.00249.x 15032839

[B2] Bari R. Jones J. D. (2009). Role of plant hormones in plant defence responses. Plant Mol. Biol. 69 (4), 473–488. doi: 10.1007/s11103-008-9435-0 19083153

[B3] Bheemanahalli R. Impa S. M. Krassovskaya I. Vennapusa A. R. Gill K. S. Obata T.. (2020). ABA and IAA-conjugate in anthers instigate heat sensitivity in spring wheat. Physiologia plantarum 169 (4), 501–514. doi: 10.1111/ppl.13109 32314362

[B4] Chen S. Cao H. Huang B. Zheng X. Liang K. Wang G. L.. (2022). The *WRKY10-VQ8* module safely and effectively regulates rice thermotolerance. Plant Cell Environ. 45 (7), 2126–2144. doi: 10.1111/pce.14329 35394666

[B5] Chen L. Sun H. Wang F. Yue D. Shen X. Sun W.. (2020). Genome-wide identification of MAPK cascade genes reveals the *GhMAP3K14*-*GhMKK11*-*GhMPK3*1 pathway is involved in the drought response in cotton. Plant Mol. Biol. 103 (1-2), 211–223. doi: 10.1007/s11103-020-00986-0 32172495

[B6] Chen Y. Yue L. J. Yang Q. Zhang H. L. Ke G. H. Liu Y. H. (2019). Effects of high temperature and heat damage on growth and development of maize and its research progress. Tillage Cultivation 01), 26–31+39. doi: 10.13605/j.cnki.52-1065/s.2019.01.008

[B7] Choi H. Hong J. Ha J. Kang J. Kim S. Y. (2000). ABFs, a family of ABA-responsive element binding factors. J. Biol. Chem. 275 (3), 1723–1730. doi: 10.1074/jbc.275.3.1723 10636868

[B8] Chopra R. Burow G. Hayes C. Emendack Y. Xin Z. Burke J. (2015). Transcriptome profiling and validation of gene based single nucleotide polymorphisms (SNPs) in sorghum genotypes with contrasting responses to cold stress. BMC Genomics 16, 1040. doi: 10.1186/s12864-015-2268-8 26645959 PMC4673766

[B9] Das A. Eldakak M. Paudel B. Kim D. W. Hemmati H. Basu C.. (2016). Leaf proteome analysis reveals prospective drought and heat stress response mechanisms in soybean. BioMed. Res. Int. 2016), 6021047. doi: 10.1155/2016/6021047 27034942 PMC4808539

[B10] Deng M. Wang Y. Kuzma M. Chalifoux M. Tremblay L. Yang S.. (2020). Activation tagging identifies *Arabidopsis* transcription factor *AtMYB68* for heat and drought tolerance at yield determining reproductive stages. Plant J. 104 (6), 1535–1550. doi: 10.1111/tpj.15019 33048399

[B11] Ding Y. Ma Y. Liu N. Xu J. Hu Q. Li Y.. (2017). MicroRNAs involved in auxin signaling modulate male sterility under high temperature stress in cotton (*Gossypium hirsutism*). Plant J. 91, 977–994. doi: 10.1111/tpj.13620 28635129

[B12] El-Esawi M. A. Al-Ghamdi A. A. Ali H. M. Ahmad M. Ahmad M. (2019). Overexpression of *AtWRKY30* transcription factor enhances heat and drought stress tolerance in wheat (*Triticum aestivum* l.). Genes 10 (2), 163. doi: 10.3390/genes10020163 30791662 PMC6410048

[B13] Fang C. Ma Y. M. Wu S. W. Liu Z. Wang Z. Yang R.. (2017). Genome-wide association studies dissect the genetic networks underlying agronomical traits in soybean. Genome Biol. 18, 161. doi: 10.1186/s13059-017-1289-9 28838319 PMC5571659

[B14] Foyer C. H. Noctor G. (2009). Redox regulation in photosynthetic organisms: signaling, acclimation, and practical implications. Antioxidants Redox Signaling 11 (4), 861–905. doi: 10.1089/ars.2008.2177 19239350

[B15] Han Y. H. Zheng Y. Z. Li T. Gao Y. (2001). Preliminary report on cumulative effects of high temperature / osmotic stress on some physiological responses of soybean. Soybean Sci. 01), 41–44.

[B16] Hou Z. Chen Q. Zhao M. Huang C. Wu X. (2020). Genome-wide characterization of the Zn(II)2Cys6 zinc cluster-encoding gene family in pleurotus ostreatus and expression analyses of this family during developmental stages and under heat stress. PeerJ 8, e9336. doi: 10.7717/peerj.9336 32566411 PMC7295025

[B17] Larkindale J. Vierling E. (2008). Core genome responses involved in acclimation to high temperature. Plant Physiol. 146 (2), 748–761. doi: 10.1104/pp.107.112060 18055584 PMC2245833

[B18] Le M. B. Poage M. Shiel K. Nugent G. D. Dix P. J. (2011). Tobacco chloroplast transformants expressing genes encoding dehydroascorbate reductase, glutathione reductase, and glutathione-s-transferase, exhibit altered anti-oxidant metabolism and improved abiotic stress tolerance. Plant Biotechnol. J. 9 (6), 661–673. doi: 10.1111/j.1467-7652.2011.00611.x 21450042

[B19] Li S. Fu Q. Chen L. Huang W. Yu D. (2011). Arabidopsis thaliana *WRKY25*, *WRKY26*, and *WRKY33* coordinate induction of plant thermotolerance. Planta 233 (6), 1237–1252. doi: 10.1007/s00425-011-1375-2 21336597

[B20] Li J. J. Nadeem M. Chen L. Y. Wang M. H. Wan M. Y. Qiu L. J.. (2020). Differential proteomic analysis of soybean anthers by iTRAQ under high-temperature stress. J. Proteomics 229, 103968. doi: 10.1016/j.jprot.2020.103968 32911126

[B21] Li C. Y. Peng C. H. Zhao Q. B. Xie P. Zhan W. (2004). Analysis on the characteristics of abnormal high temperature in the midsummer of 2003 in wuhan. J. Cent. China Normal Univ. 03, 379–382.

[B22] Li F. Zhan D. Xu L. Han L. Zhang X. (2014a). Antioxidant and hormone responses to heat stress in two Kentucky bluegrass cultivars contrasting in heat tolerance. J. Am. Soc. Hortic. Sci. Am. Soc. Hortic. Sci. 139, 587–596. doi: 10.21273/JASHS.139.5.587

[B23] Li Y. H. Zhou G. Y. Ma J. X. Jiang W. Jin L-. G. Zhang Z.. (2014b). *De novo* assembly of soybean wild relatives for pan-genome analysis of diversity and agronomic traits. Nat. Biotechnol. 32, 1045–1052. doi: 10.1038/nbt.2979 25218520

[B24] Lin H. H. Lin K. H. Syu J. Y. Tang S. Y. Lo H. F. (2016). Physiological and proteomic analysis in two wild tomato lines under waterlogging and high temperature stress. J. Plant Biochem. Biotechnol. 25, 87–96. doi: 10.1007/s13562-015-0314-x

[B25] Liu Y. H. Li J. J. Zhu Y. L. Zhang Y. P. Jones A. Rose R. J.. (2019). Heat stress in legume reproduction: effects, causes and future prospects. Front. Plant Sci. 10. doi: 10.3389/fpls.2019.00938 PMC6684746 31417579

[B26] Ma Y. Min L. Wang J. Li Y. Wu Y. Hu Q.. (2021). A combination of genome-wide and transcriptome-wide association studies reveals genetic elements leading to male sterility during high temperature stress in cotton. New Phytol. 231 (1), 165–181. doi: 10.1111/nph.17325 33665819 PMC8252431

[B27] Malik S. I. Hussain A. Yun B. W. Spoel S. H. Loake G. J. (2011). GSNOR-mediated de-nitrosylation in the plant defence response. Plant Sci. 181 (5), 540–544. doi: 10.1016/j.plantsci.2011.04.004 21893250

[B28] Mizoguchi T. Irie K. Hirayama T. Hayashida N. Yamaguchi-Shinozaki K. Matsumoto K.. (1996). A gene encoding a mitogen-activated protein kinase kinase kinase is induced simultaneously with genes for a mitogen-activated protein kinase and an S6 ribosomal protein kinase by touch, cold, and water stress in arabidopsis thaliana. Proc. Natl. Acad. Sci. United States America 93 (2), 765–769. doi: 10.1073/pnas.93.2.765 PMC40129 8570631

[B29] Muhammad N. Li J. J. Wang M. H. Shah L. Lu S. Wang X. B. (2018). Unraveling field crops sensitivity to heat stress: mechanisms, approaches, and future prospects. Agronomy 8 (7), 128. doi: 10.3390/agronomy8070128

[B30] Oberholster S. Peterson C. Dute R. (1991). Pedicel abscission of soybean: cytological and ultrastructural changes induced by auxin and ethephon. Can. J. Bot. 69, 2177–2186. doi: 10.1139/b91-273

[B31] Pradhan S. K. Pandit E. Nayak D. K. Behera L. Mohapatra T. (2019). Genes, pathways and transcription factors involved in seedling stage chilling stress tolerance in indica rice through RNA-seq analysis. BMC Plant Biol. 19 (1), 352. doi: 10.1186/s12870-019-1922-8 31412781 PMC6694648

[B32] Raju G. Shanmugam K. Kasirajan L. (2020). High-throughput sequencing reveals genes associated with high-temperature stress tolerance in sugarcane. 3 Biotech. 10 (5), 198. doi: 10.1007/s13205-020-02170-z PMC7148410 32300514

[B33] Ren C. Bilyeu K. D. Beuselinck P. R. (1999). Composition, vigor, and proteome of mature soybean seeds developed under HT. Crop Sci. 49 (3), 1010–1022. doi: 10.2135/cropsci2008.05.0247

[B34] Ren Y. Huang Z. Jiang H. Wang Z. Wu F. Xiong Y.. (2021). A heat stress responsive NAC transcription factor heterodimer plays key roles in rice grain filling. J. Exp. Bot. 72 (8), 2947–2964. doi: 10.1093/jxb/erab027 33476364

[B35] Rizhsky L. Liang H. Mittler R. (2002). The combined effect of drought stress and heat shock on gene expression in tobacco. Plant Physiol. 130 (3), 1143–1151. doi: 10.1104/pp.006858 12427981 PMC166635

[B36] Sakata T. Higashitani A. (2008). Male Sterility accompanied with abnormal anther development in plants–genes and environmental stresses with special reference to high temperature injury. Int. J. Plant Dev. Biol. 2, 42–51.

[B37] Sang Q. Q. Shan X. An Y. H. Guo S. R. Sun J. (2017). Proteomic analysis reveals the positive effect of exogenous spermidine in tomato seedlings response to high-temperature stress. Frontier Plant Science. 8. doi: 10.3389/fpls.2017.00120 PMC5292424 28220137

[B38] Shi Z. J. Chen Y. T. Xu Y. Ji D. H. Chen C. S. Xie C. T. (2017). Differential proteomic analysis by iTRAQ reveals the mechanism of pyropia haitanensis responding to high temperature stress. Sci. Rep. 7, 44734. doi: 10.1038/srep44734 28303955 PMC5356179

[B39] Suzuki N. Miller G. Morales J. Shulaev V. Torres M. A. Mittler R. (2011). Respiratory burst oxidases: the engines of ROS signaling. Curr. Opin. Plant Biol. 14 (6), 691–699. doi: 10.1016/j.pbi.2011.07.014 21862390

[B40] Tian X. H. Luo H. W. Zhou H. D. Wu C. Y. (2009). High temperature stress on rice anthesis: research progress and prospects. Chin. J. Appl. Ecol. 25 (22), 166–168. doi: 10.11924/j.issn.1000-6850.2009-1644 18260475

[B41] Timabud T. Yin X. J. Pongdontri P. Komatsu S. (2016). Gel-free/label-free proteomic analysis of developing rice grains under heat stress. J. Proteome 133, 1–19. doi: 10.1016/j.jprot.2015.12.003 26655677

[B42] Wang J. Xu J. Wang L. Zhou M. Nian J. Chen M.. (2023). SEMI-ROLLED LEAF 10 stabilizes catalase isozyme b to regulate leaf morphology and thermotolerance in rice (*Oryza sativa* l.). Plant Biotechnol. J. 21 (4), 819–838. doi: 10.1111/pbi.13999 36597711 PMC10037157

[B43] Wang M. Yuan J. Qin L. Shi W. Xia G. Liu S. (2020b). *TaCYP81D5*, one member in a wheat cytochrome P450 gene cluster, confers salinity tolerance *via* reactive oxygen species scavenging. Plant Biotechnol. J. 18 (3), 791–804. doi: 10.1111/pbi.13247 31472082 PMC7004906

[B44] Wang B. Zhong Z. Wang X. Han X. Yu D. Wang C.. (2020a). Knockout of the *OsNAC006* transcription factor causes drought and heat sensitivity in rice. Int. J. Mol. Sci. 21 (7), 2288. doi: 10.3390/ijms21072288 32225072 PMC7177362

[B45] Xu X. Y. Shao C. X. Sun Z. G. Long B. J. Dong W. L. (2021). Research progress on the effect of heat stress on physiological characteristics of maize at key growth stage and the yield. J. Maize Sci. 29 (02), 81–88+96. doi: 10.32615/ps.2021.060

[B46] Yang S. Cai W. Shen L. Cao J. Liu C. Hu J.. (2022). *CaCDPK29*–*CaWRKY27b* module promotes *CaWRKY40*-mediated thermotolerance and immunity to ralstonia solanacearum in pepper. New Phytol. 233 (4), 1843–1863. doi: 10.1111/nph.17891 34854082

[B47] Zhang G. Guo G. Hu X. Zhang Y. Li Q. Li R.. (2010). Deep RNA sequencing at single base-pair resolution reveals high complexity of the rice transcriptome. Genome Res. 20 (5), 646–654. doi: 10.1101/gr.100677.109 20305017 PMC2860166

[B48] Zhang Y. F. Lou H. Y. Guo D. D. Zhang R. Q. Zheng M. S. Hou H.. (2018). Identifying changes in the wheat kernel proteome under heat stress using iTRAQ. Crop J. 4, 11. doi: 10.1016/j.cj.2018.04.003

[B49] Zhao C. Liu B. Piao S. L. Wang X. H. Lobell D. B. Huang Y.. (2017a). Temperature increase reduces global yields of major crops in four independent estimates. Proc. Natl. Acad. Sci. United States America 114 (35), 9326–9331. doi: 10.1073/pnas.1701762114 PMC5584412 28811375

[B50] Zhao Y. Tian X. J. Wang F. Zhang L. Xin M. M. Hu Z. R.. (2017b). Characterization of wheat MYB genes responsive to high temperatures. BMC Plant Biol. 17 (1), 208. doi: 10.1186/s12870-017-1158-4 29157199 PMC5696766

[B51] Zhou Y. Wang Y. Xu F. Song C. Yang X. Zhang Z.. (2022). Small HSPs play an important role in crosstalk between HSF-HSP and ROS pathways in heat stress response through transcriptomic analysis in lilies (*Lilium longiflorum*). BMC Plant Biol. 22 (1), 202. doi: 10.1186/s12870-022-03587-9 35439940 PMC9017035

[B52] Zong X. J. Li D. P. Gu L. K. Li D. Q. Liu L. X. Hu X. L. (2009). Abscisic acid and hydrogen peroxide induce a novel maize group c MAP kinase gene, *ZmMPK7*, which is responsible for the removal of reactive oxygen species. Planta 229 (3), 485–495. doi: 10.1007/s00425-008-0848-4 19002491

