# Supplementary figures and images for: Integrated transcriptome and proteome analysis reveals molecular responses of soybean anther under high-temperature stress
Source: Front Plant Sci. 2023 Jun 14;14:1187922. doi: 10.3389/fpls.2023.1187922 (PMC10303809; doi:10.3389/fpls.2023.1187922)

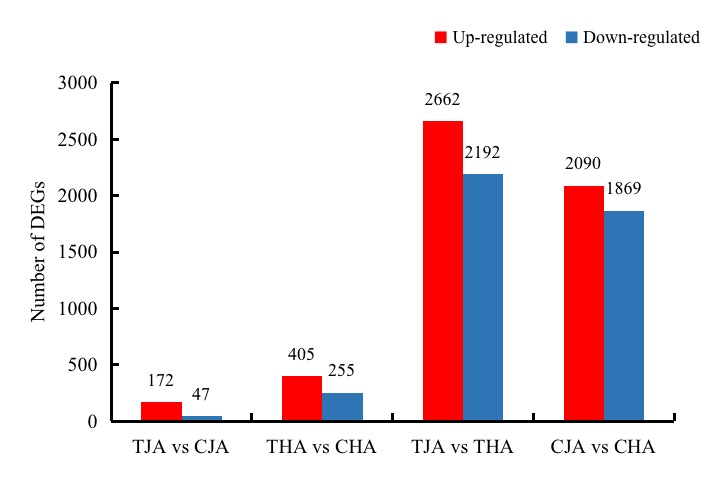

Supplement: Supplementary Figure 1 — The number of differentially expressed genes between TJA vs CJA, THA vs CHA, TJA vs THA and CJA vs CHA. [file Image_1.jpeg]
